# Reassessing the level and implications of male involvement in family planning in Indonesia

**DOI:** 10.1186/s12905-023-02354-8

**Published:** 2023-05-03

**Authors:** Sukma Rahayu, Nohan Arum Romadlona, Budi Utomo, Riznawaty Imma Aryanty, Elvira Liyanto, Melania Hidayat, Robert J. Magnani

**Affiliations:** 1 Knowledge Hub for Reproductive Health, Faculty of Public Health, Universitas Indonesia, Kota Jakarta Pusat, Indonesia; 2 UNFPA, Kota Jakarta Pusat, Indonesia; 3 Department of Public Health Science, Faculty of Sport Science, Universitas Negeri Malang, Kota Malang, Indonesia

**Keywords:** Indonesia, Male involvement, Family planning

## Abstract

**Background:**

Although there is global recognition of the importance of involving men in family planning and reproductive health matters, this issue has received insufficient attention in many countries. The present study sought to characterize married Indonesian males as to their level of involvement in family planning, identify the correlates thereof and assess the implications of male involvement for unmet need for family planning.

**Methods:**

A mixed methods research design was used. The main source of quantitative data was 2017 Indonesian Demographic Health Survey (IDHS) data from 8,380 married couples. The underlying “dimensions” of male involvement were identified via factor analysis. The correlates of male involvement were assessed via comparisons across the four dimensions of male involvement identified in the factor analysis. Outcomes were assessed by comparing women’s and couple’s unmet need for family planning for the four underlying dimensions of male involvement. Qualitative data were collected via focus group discussions with four groups of key informants.

**Results:**

Indonesian male involvement as family planning clients remains limited, with only 8% of men using a contraceptive method at the time of the 2017 IDHS. However, factor analyses revealed three other independent “dimensions” of male involvement, two of which (along with male contraceptive use) were associated with significantly lower odds of female unmet need for family planning. Male involvement as clients and passive male approval of family planning, which in Indonesia empowers females take action to avoid unwanted pregnancies, were associated with 23% and 35% reductions in female unmet need, respectively. The analyses suggest that age, education, geographic residence, knowledge of contraceptive methods, and media exposure distinguish men with higher levels of involvement. Socially mandated gender roles concerning family planning and perceived limited programmatic attention to males highlight the quantitative findings.

**Conclusions:**

Indonesian males are involved in family planning in several ways, although women continue to bear most of the responsibility for realizing couple reproductive aspirations. Gender transformative programming that addresses broader gender issues and targets priority sub-groups of men as well as health service providers, community and religious leaders would seem to be the way forward.

## Abstract (summary)

Despite global recognition of the importance of involving men in family planning and women’s health matters, this matter has received insufficient the attention in many countries. The present study sought to characterize married Indonesian males as to their level of involvement in family planning, identify correlates of male involvement, and assess the implications of varying levels of male participation for family planning outcomes.

The study used a mixed methods research design. The main source of quantitative data was a 2017 Indonesian Demographic Health Survey (IDHS) data set of 8,380 married couples. Factor analyses were undertaken to identify the underlying dimensions of male involvement. The correlates of male involvement were assessed via comparisons across the four underlying dimensions of male involvement group identified in the factor analysis. Outcomes were assessed by comparing women’s and couple’s unmet need for family planning for the four underlying dimensions of male involvement. Qualitative data were collected via focus group discussions with four groups of key informants.

The study found that male involvement as family planning clients remains limited, with only 8% of men using contraceptive methods themselves. However, Indonesian men are involved in other ways such via approval of family planning and active communications that contribute to lower female unmet need for family planning. The analyses suggest that age, education, geographic residence, knowledge of contraceptive methods, and media exposure distinguish men with higher versus lower levels of involvement. The most important contribution of males to realizing couple-level desires to limit or space births is via the approval of family planning, which empowers females take action to avoid unwanted pregnancies. Socially mandated gender roles concerning family planning and perceived limited programmatic attention to males are highlighted in the quantitative findings.

In the way of an overall conclusion, Indonesian males are involved in family planning in several ways, although women continue to bear most of the responsibility for realizing couple reproductive aspirations. Gender transformative programming that addresses broader gender issues and targets priority sub-groups of men as well as health service providers, community and religious leaders would seem to be the way forward.

## Background

Interest in men’s roles in family planning data back to at least the 1980s [1]. A global consensus as to the importance of involving men in family planning and broader reproductive health matters was reached at the 1994 International Conference on Population and Development in Cairo (ICPD) [2–4]. A sizeable literature has since been amassed making the case that men constitute an important asset both in the realization of reproductive aspirations and in efforts to improve women’s health [1–10], documenting the determinants of male involvement [11–24], assessing the impact of male participation (or lack thereof) on reproductive health outcomes [22, 25–35], and assessing the progress being made [36–38]. Engaging men and boys in family planning is included among the high impact practices (HIPs) for family planning [39] and the Male Contraceptive Initiative [40] has elaborated on how increased attention to male contraception could contribute to the realization of each of the 17 Sustainable Development Goals (SDGs).

Although Indonesia’s National Family Planning Program (NFP) is generally viewed as having been successful, the program has experienced some degree of stagnation in the past two decades. The contraceptive prevalence rate (CPR) increased only from 60.3% to 2002 to 63.6% in 2017 and the modern contraceptive prevalence rate (mCPR) from 56.7% to 2002 to only 57.2% in 2017 [41]. The level of demand for family planning (i.e., fecund women of reproductive age who desire no further children or to delay their next child by two years or more) grew during this period from 69.7% to 2002 to 74.2% in 2017, but the level of unmet need for family planning also increased (from 8.6% to 2002 to 10.6% in 2017).

One issue that has perhaps not received sufficient attention in trying to revive the NFP is that of male involvement in family planning. While national reproductive health/family planning policy clearly establishes optimizing the reproductive health of families, like many national family planning programs the Indonesian NFP has over the years focused programmatic attention primarily on women. Indeed, Hull [41] observed many years ago Indonesia missed an opportunity early on to promote use of male methods. Collectively, researchers have attributed insufficient attention to male involvement to three primary factors: [1] Emphasis on clinical service delivery to women of reproductive age and on women’s barriers to contraceptive use, [2] Assumptions about open communication and evenly shared reproductive decisions between men and women, and [3] Lack of research on men’s attitudes and behaviors and gaps in evaluation data for interventions on men.

Greene et al. [2] propose that the male involvement in family planning may be viewed as falling into three domains: men as family planning clients, men as partners, and men as agents of change. The participation of married men as clients in Indonesia remains limited. Among currently married women who reported contracepting at the time of the 2017 Indonesian Demographic and Health Surveys (IDHS) [42], only 8% were relying on “male controlled” methods – 1.5% vasectomy, 36.7% male condoms, and 61.9% withdrawal. However, outright opposition to family planning among Indonesian men is also limited – per the 2017 IDHS, only 4.3% of husbands in the “couples sample” disapproved of family planning. The key to identifying potential program opportunities to strengthen the NFP via increased male involvement thus lies in acquiring a deeper understanding of the vast majority of married Indonesian men who are neither family planning clients nor opponents of family planning in terms of their reproductive aspirations, perspectives on male involvement in family planning decision making, and actual levels of and barriers to increased involvement. To date no such systematic, in-depth assessment has been undertaken. The existing Indonesian studies on these matters are either dated (i.e., based upon data from 2010 or earlier), focus on particular local settings, or were not peer reviewed [32].

The present study sought to characterize the male spouses of currently married Indonesian women with regard to their level of involvement in family planning, identify the correlates of different levels of male involvement, and assess the implications of varying levels of male participation for family planning outcomes in order to better understand the key “levers” that might be used by the NFP to increase male involvement in family planning. The study was undertaken from the perspective of married couples since understanding the family planning-related actions of men requires knowledge of the reproductive aspirations of both marital partners, which may or may not be the same. The need for couples-level analyses was recognized many years ago by Stan Becker [43], who developed an algorithm for measuring unmet need for family planning among couples. Although this conceptual insight has subsequently received relatively little attention, we believe that it is crucial to understanding male involvement in family planning.

## Methods and materials

A mixed methods research design was used in the study featuring both quantitative and qualitative data. The main source of data for the quantitative portion of the study was the 2017 Indonesian Demographic Health Survey (IDHS). We created a “couples” dataset consisting of responses to the female and married male IDHS questionnaires for 8,380 married couples.

Three [3] forms of quantitative analyses were undertaken. All analyses were limited to couples in which the female partners were fecund. First, as background for the remainder of the study we assessed the degree of concordance (or lack thereof) in terms of demand for family planning (i.e., desire to limit or space future pregnancies) and the contraceptive behaviors of both partners associated with partner combinations. Second, we examined variables measured in the 2017 IDHS that depicted male involvement in family planning. These consisted of a combination of attitudes, behaviors, and future expectations. The variables considered were:

- Approve of FP.
- Discussed FP with wife in last year.
- Discussed FP with other persons in the last 6 months.
- Involved in decisions whether wife should/should not use contraception.
- Disagreed with the statement that “Contraception is women’s business; men should not worry”.
- Disagreed with the statement that “A woman is the one who gets pregnant, so she should be the one to get sterilized”.
- Using a contraceptive method at the time of the survey.
- Expect to use a method in the future.

After documenting the level/prevalence of these variables in the full sample of couples, we then assessed the covariates or correlates of these indicators of male involvement. The factors considered included socioeconomic background factors, geographic residence, and exposure to family planning program interventions. We then assessed whether the eight variables “hung together” such that a meaningful male involvement scale could be produced. However, factor analysis/principal components analysis indicated that there were four [4] independent underlying dimensions of male involvement in family planning in Indonesia (details provided below in the Results section). Accordingly, the factor scores for sample couples on these four dimensions were used as the measure of male involvement in subsequent analyses. We assessed the covariates or correlates of the four underlying dimensions of male involvement with regard to socioeconomic and geographic residence background factors, and exposure to family planning program interventions via ordinary least squares (OLS) regression.

The final step in the quantitative analysis was to assess the relationship between dimensions of male family planning involvement identified in the factor analysis and unmet for family planning for both females and couples. Demand for family planning was defined in the case of women as women desiring to avoid further births or wanting to delay their next birth by at least two years. Couple-level demand was defined as either spouse desiring to limit or space further births. Unmet need for family planning in the case of women was defined as women with demand for family planning but neither she nor her spouse were using a contraceptive method at the time of the 2017 IDHS. Unmet need for couples was defined as either spouse having demand for family planning and neither were using a contraceptive method. Both unmet need calculations included women and couples in which the women was pregnant or breastfeeding at the time of the 2017 IDHS using data on the wanted status of the current pregnancy or most recent birth to define unmet need from the female perspective. Couples in which women whose current pregnancy was either mistimed or unwanted (from their own perspective) were classified as having unmet need, as were couples in which the most recent birth of women who were breastfeeding at the time survey were reported as having been either mistimed or unwanted (again from female perspective). This procedure will understate the actual level of couple’s unmet need as it does not consider current pregnancies/recent births that were wanted by the wife of married couples but not the husband. This bias is unavoidable since husbands were not asked about the wanted status of current pregnancies/recent births in the 2017 IDHS. However, it is likely to be small in magnitude as cases of discrepant demand for family planning within couples in which the male had demand for family planning and the female did not is relatively rare among married Indonesian couples in which the wife was neither pregnant nor breastfeeding at the time of the 2017 IDHS (see Table 1 in the Results section).

Qualitative data were collected from four [4] groups of key stakeholders via virtual focus group discussions (FGDs) from August 12–18, 2021. Members of the following key stakeholder groups were recruited as FGDs participants: married men of reproductive age (23 participants), married women of reproductive age (24 participants), program managers (24 participants), community and religious leaders (24 participants). Equal numbers of participants were chosen from National Population and Family Planning Board BKKBN priority districts in each of the three major regions of Indonesia (western, central, and eastern Indonesia) so as to ensure geographic diversity. FGD participants were chosen by staff at local BKKBN offices and advised on when to come to the office to participate in the FGDs. Gender perspectives were obtained on the following matters via the FGDs with married men and women: [1] knowledge of family planning, [2] family planning information, [3] gender roles in and decision-making regarding family planning, [4] decision making regarding number of children, [5] family planning experience, and [6] barriers to men using family planning. The FGDs with program managers focused on: [1] financing male family planning, [2] adequacy of service providers, [3] training, [4] socio-cultural factors influencing uptake of male family planning, [5] government programs, [6] perceptions on male family planning participation, and [7] geographic access issues. The FGDs with community and religious leaders focused on: [1] perspectives on family planning and family planning programs, [2] influence of sociocultural and traditional beliefs, [3] role of religious institutions, [4] forms of collaboration with family planning programs, [5] barriers to supporting family planning programs, and [6] barriers to men using family planning. All discussions were recorded and transcribed verbatim. After validating the transcripts, the narratives were translated into English and verified for accuracy by a native speaker. Analysis of the data was conducted using thematic content analysis and included several iteratives steps. Direct quotations from men, women, program managers and community and religious leaders are presented in italics to highlight key findings.

We first present the quantitative results, followed by insights and perspectives provided by the qualitative data. Insights from the quantitative and qualitative analyses are then synthesized in the Discussion section of the manuscript.

## Results

Couple-level data on demand for family planning and contraceptive use at the time of the.

2017 IDHS the are displayed in Table 1. Overall, the level of demand for family planning was 74% among females and 66% among males. Concordance in demand for family planning is observed for the large majority (75%) of couples – 58% both wanting to limit or space future births and 17% neither wanting to limit or space. Among the 23% of couples whose demand for family planning was discordant, cases of females wanting to limit of space while their spouse did not aspire to do so were twice as common (16%) as discordant cases in which it was the male that wanted to limit or space, and the female did not (8%).

Table 1 also documents the contraceptive behaviors of both marital partners at the time of the survey in relation to couple-level demand for family planning. Several observations emerge from these data. First, male contraceptive use appears to be largely unresponsive to variations in either male or couple-level demand for family planning with only minor variations observed around low levels of male contraceptive use. Female contraceptive behavior on the other hand appears to be directly responsive to female demand to limit or space births as well as to satisfy male demand – 60% of women who did not want to limit or space future births but whose husbands did were using a contraceptive method at the time of the 2017 IDHS. Second, there appears to be substantial level of contraceptive use even in the absence of a desire to limit or space births by either marital partner. 30% of couples without apparent demand for family planning were using a contraceptive method at the time of the 2017 IDHS – 25% of females and 2% of males, with 2% of couples practicing redundant contraception. 39% of these couples were either uncertain about wanting more children or were not sure when, while 61% wanted a child within two years and may have been contracepting to delay the pregnancy. The redundant use of contraceptive methods by marital partners is also noteworthy. In virtually all cases this entailed males using condoms or withdrawal to supplement contraceptive use by their wives.

**Table 1:** Summary of Demand for Family Planning and Contraceptive Behaviors of Currently Married Indonesian Couples *

Couple Aspirations	Number	Percent	Percent Using a Contraceptive Method			
			Wife	Husband	Both	Neither
Wife wants to limit or space, husband no	922	16%	80%	3%	3%	14%
Husband wants to limit or space, wife no	481	8%	60%	3%	4%	33%
Both want to limit or space	3,383	58%	80%	4%	6%	10%
Neither want to limit or space	1,006	17%	25%	2%	2%	70%
Total	5,792					

* Excludes couples in which the wife was infecund, pregnant, or breastfeeding at the time of the 2017 IDHS.

**Fig. 1:**
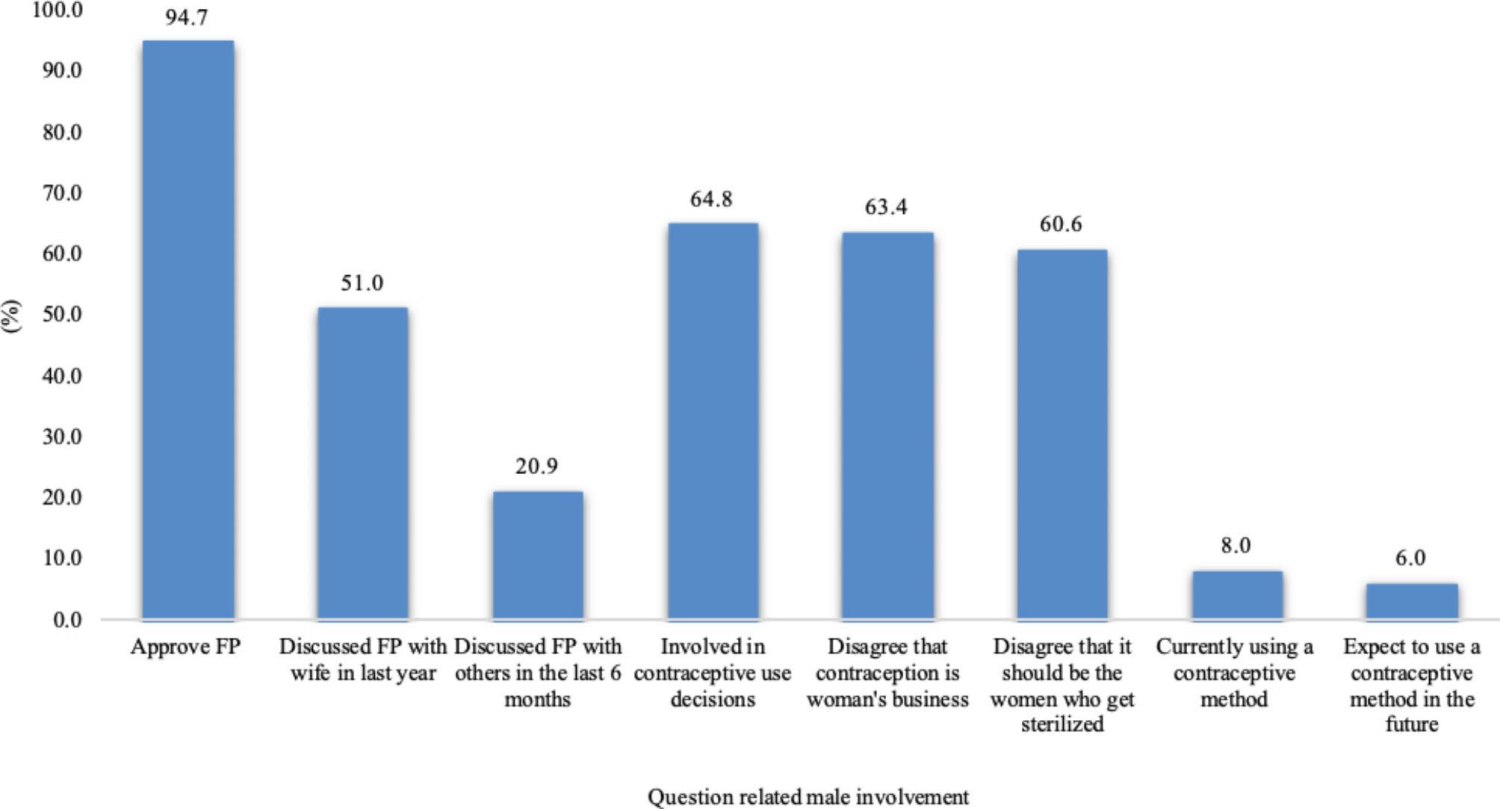
Levels of Male Involvement by Type of Involvement

Eight variables extracted from the 2017 IDHS were used in the study to characterize men’s involvement in family planning in Indonesia (see Methods section for details). The levels or prevalence of male involvement indicated by these variables are documented in Fig. 1. As may be observed, Indonesian men overwhelmingly approve of family planning and about 65% have been involved in contraceptive use/non-use decisions. 61% of men had discussed family planning with their wife in the 12 months prior to the 2017 IDHS and 21% had discussed family planning with others in the six months prior to the survey. A majority of men did not subscribe to the view that family planning was the sole responsibility of the female. However, involvement as family planning clients was relatively low – only eight [8] percent were using a male-controlled contraceptive method at the time of the 2017 IDHS and six [6] percent of men who were not using a male-controlled method at the time of the survey expected to use such methods in the future.

Table 2 presents data on the sociodemographic and residential correlates of the male involvement indicators considered in the study. As may be observed, the indicators tend to vary systematically by socioeconomic level and residence. The exception is approval of family planning, which is close to universal. For all other indicators, men with higher levels of education, greater household wealth, and with professional or clerical occupations were more likely to be involved in family planning. The same was true of men residing in urban vs. rural areas and for the most part men residing on Java-Bali vs. those residing on outer islands. However, men residing on Java-Bali vs. outer islands did not differ on male contraceptive use or expectations to use contraceptive methods in the future. Younger men showed higher levels of involvement than older men with the exception of approval of family planning and current contraceptive use.

**Table 2:** Background Factor Correlates of Male Involvement in Family Planning

Background	Approve FP	Discussed FP with wife in last year	Discussed FP with others in the last 6 months	Disagree that contraception is women’s business	Involved in decision contraceptive use decision	Disagree that it should be the women who get sterilized	Currently using a contraceptive method	Expected to use a contraceptive method in the future
**Age**								
< 30	92.1*	60.5**	27.1**	62.3 (ns)	68.7*	59.8 (ns)	5.5**	11.1**
30–40	94.8	56.2	22.4	63.9	66.2	59.7	7.4	7.5
> 40	95.4	42.4	17.3	63.2	62.3	61.9	9.5	3.9
**Education**								
No education & primary	95.3 (ns)	42.0**	13.6**	49.9**	58.7**	54.4**	4.7**	3.3**
Secondary	94.8	54.6	22.1	66.7	66.2	61.6	8.5	7.6
Higher	92.9	60.0	36.1	86.4	76.3	73.4	15.0	10.9
**Occupation**								
Not working	91.3*	54.3**	33.7**	75.7**	51.8**	61.0**	6.4**	5.7**
Professional	91.8	56.3	32.4	80.6	73.1	71.5	14.0	11.8
Clerical	95.8	59.5	36.8	79.6	77.0	69.7	12.6	11.6
Sales	95.9	50.3	20.2	65.2	64.8	64.5	8.0	6.7
Self-employ	94.4	41.6	14.7	53.0	62.8	49.8	5.1	4.3
Employee	95.2	53.5	18.3	63.4	63.6	62.4	7.7	5.7
Services	94.7	55.7	24.1	63.0	63.1	62.3	8.5	7.1
**Wealth index**								
Poorest	92.9 (ns)	45.7*	16.0**	54.3**	62.6*	53.9**	5.3**	7.2(ns)
Poorer	94.9	50.8	17.0	56.3	62.9	56.0	7.2	6.0
Middle	95.5	52.8	20.4	65.8	65.1	62.0	7.7	5.7
Richer	94.6	50.8	21.4	65.0	63.8	63.7	7.3	6.7
Richest	95.3	53.6	28.8	73.4	69.3	65.6	12.0	7.2
**Region**								
Java-Bali	95.7**	54.2**	22.1*	66.6**	62.2**	67.0**	7.7 (ns)	6.1 (ns)
Outside Java-Bali	93.3	45.9	19.2	58.4	68.9	50.5	8.4	7.3
**Residence**								
Urban	94.4 (ns)	53.4**	22.7**	70.4**	65.4 (ns)	65.7**	10.2**	7.4*
Rural	95.0	48.6	19.3	56.7	64.3	55.7	5.9	5.7
**Total**	**94.7**	**51.0**	**20.9**	**63.4**	**64.8**	**60.6**	**8.0**	**6.5**

(ns) not significant; * p value < 0.05 ; ** p value < 0.001.

Table 3 presents data on the correlates of varying levels of male involvement related to exposure to family planning interventions. As may be observed, the strength of association of program variables with level of male involvement was generally lower than that for the sociodemographic variables. The most sizeable and statistically significant differences observed were for the Method Knowledge and Media Exposure variables, with men with greater method knowledge and media exposure being consistently more likely to be involved in family planning across the set of male involvement indicators considered. Only small and often not statistically significant differences are observed to the other program exposure variables considered.

**Table 3:** Program Factor Correlates of Male Involvement in Family Planning

Background	Approve FP	Discussed FP with wife	Discussed FP with others	Involved in decision making	Disagree to ‘Contraceptive is women’s business’	Disagree to women’s responsibilities to be sterilized	Male using method	Expected to use method in the future
**FP info from health workers**								
No	94.7**	48.1**	19.3**	64.2 (ns)	62.3*	61.0 (ns)	8.6*	6.1 (ns)
Yes	95.6	58.5	25.2	66.5	66.1	59.4	6.3	7.6
**FP info from FP officer**								
No	94.7 (ns)	50.6 (ns)	20.3**	64.5 (ns)	63.4 (ns)	60.9 (ns)	8.1 (ns)	6.5 (ns)
Yes	95.6	55.0	28.0	68.3	63.3	56.7	6.7	7.0
**FP info from village leaders**								
No	94.7 (ns)	50.9 (ns)	20.9 (ns)	64.8 (ns)	63.3 (ns)	60.7 (ns)	8.1 (ns)	6.5 (ns)
Yes	97.0	53.1	23.8	65.5	66.2	54.9	5.7	6.8
**FP info from PKK**								
No	94.6 (ns)	50.4*	20.6 (ns)	64.1 (ns)	63.3 (ns)	60.7 (ns)	7.9 (ns)	6.7 (ns)
Yes	95.5	55.0	23.6	69.8	63.6	59.9	9.0	5.7
**Distance to health facility**								
Big problem	92.5*	46.9*	17.3*	61.5 (ns)	61.8 (ns)	60.2 (ns)	6.0 (ns)	7.9 (ns)
Not a big problem	95.0	51.4	21.3	65.2	63.5	60.6	8.2	6.4
**Method knowledge**								
Below mean	94.0*	43.2**	12.2**	60.8**	52.7**	56.6**	5.2**	4.7**
Above mean	95.4	58.2	29.1	68.5	73.3	64.2	10.6	8.2
**Media exposure**								
Low	93.4**	47.0**	17.2**	61.6**	55.9**	58.7*	5.8**	5.3**
Medium	95.7	49.4	20.6	63.4	62.3	59.9	7.0	5.9
High	94.7	56.4	24.7	69.6	71.5	63.1	11.3	8.5
**Total**	**94.7**	**51.0**	**20.9**	**64.8**	**63.4**	**60.6**	**8.0**	**6.5**

(ns) not significant; * p value < 0.05 ; ** p value < 0.001; PKK – Family Welfare Program Officer; PLKB – Community family planning worker

To assess whether the eight male involvement variables could be combined into a meaningful male involvement scale or index, a factor analysis/principal components analyses was run. The analysis, the results of which are displayed in Table 4, yielded four [4] independent dimensions of male involvement in family planning in Indonesia. Per standard practice in factor analysis, the dimensions of factors are interpreted or “named” based the variables that “load” on the respective factors. Only two [2] variables have significant loadings on Factor 1, both pertaining to discussions or communications about family planning, leading to the proposed naming of this factor as “Involvement via Consultation”. This factor is characterized by lukewarm approval for family planning and no involvement as a family planning client. Similarly, two variables have sizeable loadings on Factor 2, both pertaining to positive gender-role perceptions toward family planning, leading to the proposed naming of this factor as “Involvement via Positive Gender Role Perspective.” It will be noted that the discussion of family planning with wife and current/expected contraceptive use variables also had modest loadings on this factor. Factor 3 also has two variables with strong loadings, involvement in contraceptive use decision making and male contraceptive use and thus appears to correspond to “Active and Direct Male Involvement.” Only one variable loads on Factor 4, albeit very strongly – Approval of family planning. This dimension appears to represent “Passive Support via Approval.”

**Table 4:** Factor Analysis of Male Involvement Indicators

Variable	Factor 1	Factor 2	Factor 3	Factor 4
Approve FP	0.0170	-0.0081	0.0030	0.9909
Discussed FP with wife in last year	0.8118	0.1012	-0.0282	0.0862
Discussed FP with others in the last 6 months	0.8181	-0.0415	0.0884	-0.0494
Involved in contraceptive use decision	0.0674	-0.0250	0.7516	-0.0675
Disagree that contraception is woman’s business	0.0641	0.7703	0.1215	0.0384
Disagree that it should be the woman who get sterilized	0.0010	0.8058	-0.0548	-0.0487
Male currently using method or not using but expect to use contraceptive method in the future	0.0026	0.0882	0.7353	0.0800

To assess the correlates of the four underlying dimensions of family planning involvement, ordinary least squares (OLS) regressions were run using the same set of correlates considered above as independent or predictor variables. The results are shown in Table 5. With regard to age, the scores for older men on Factors 1 (Communications regarding family planning) and 3 (Active direct involvement) and higher scores on Factor 4 (Passive approval of family planning). Men with higher levels of education had higher scores for Factors 1–3 but not Factor 4, reflecting the fact that approval of family planning is nearly universal among Indonesian men. Occupation and household wealth were not strongly associated with any of the styles of male involvement depicted by the four factors identified in the factor analysis, although men with professional or clerical occupations had somewhat higher scores on Factor 3 (Active direct involvement). Male involvement factor scores were largely unrelated to urban-rural residence, while men residing on Java-Bali had higher factor scores for three of the four factors (all except Factor 3 – Active direct involvement). Higher scores for this factor outside of Java-Bali might reflect the need for men to be more directly involved due to the greater barriers to women sustaining contraceptive use in such locations as compared to Java-Bali.

**Table 5:** Regression of Factor Scores on Potential Correlates of Male Involvement

Background	Coefficient					
	Factor 1	Factor 2	Factor 3		Factor 4	
**Age**						
**<** 30	Ref	Ref	Ref	Ref		
30–40	-0.176**	0.012	-0.075	0.112*		
**>** 40	-0.397**	0.065	-0.100*	0.137**		
**Education**						
No education & primary	Ref	Ref	Ref	Ref		
Secondary	0.116**	0.224**	0.139**	-0.021		
Higher	0.279**	0.465**	0.319**	-0.127*		
**Wealth index**						
Poorest	Ref	Ref	Ref	Ref		
Poorer	-0.002	-0.027	-0.015	0.047		
Middle	0.007	0.105*	-0.008	0.060		
Richer	-0.049	0.055	-0.076	0.014		
Richest	-0.052	0.019	-0.0	0.068		
**Region**						
Java-Bali	Ref	Ref	Ref	Ref		
Outside Java-Bali	-0.119**	-0.272**	0.107**	-0.097**		
**Residence**						
Urban	Ref	Ref	Ref	Ref		
Rural	0.059	-0.089**	-0.036	0.047		
**Occupation**						
Not working	Ref	Ref	Ref	Ref		
Professional	-0.191	0.023	0.404**	0.052		
Clerical	-0.103	-0.002	0.419**	0.213		
Sales	-0.269	-0.077	0.257*	0.158		
Self-employ	-0.269*	-0.077	0.219	0.178		
Employee	-0.256*	-0.052	0.258*	0.176		
Services	-0.147	-0.109	0.243	0.154		
**FP info from health workers**						
No	Ref	Ref	Ref	Ref		
Yes	0.156**	-0.003	-0.064	0.109**		
**FP info from FP officer**						
No	Ref	Ref	Ref	Ref		
Yes	0.097	-0.043	-0.019	-0.018		
**FP info from village leaders**						
No	Ref	Ref	Ref	Ref		
Yes	-0.024	-0.003	-0.063	0.068		
**FP info from PKK**						
No	Ref	Ref	Ref	Ref		
Yes	-0.018	-0.057	0.119*	-0.062		
**Distance to health facility**						
Big problem	Ref	Ref	Ref	Ref		
Not a big problem	0.035	-0.108*	0.005	0.084		
**Method knowledge**						
Below mean	Ref	Ref	Ref	Ref		
Above mean	0.344**	0.161**	0.149**	0.073**		
**Media exposure**						
Low	Ref	Ref	Ref	Ref		
Medium	-0.004	0.023	0.046	0.082*		
High	-0.005	0.036	0.175**	0.050		
**Cons**	**0.173**	**-0.003**	**-0.486**	**-0.454**		

* p value < 0.05 ; ** p value < 0.001

What is the impact of male involvement on unmet for family planning for both women and couples? Table 6 displays the odds ratios of unmet need (from logistic regressions) for differing levels of male involvement as measured by factor scores on the four dimensions of male involvement identified in the earlier factor analysis. These results indicate that most forms of male involvement in family planning lead to lower levels of unmet need among women. High factor scores on Factor 1 (Active Consultation regarding Family Planning) results in 10% lower odds of female unmet need. The corresponding figures for Factors 3 (Active and Direct Involvement) and 4 (Approval of Family Planning, Passive) are 22% and 35% lower odds of female unmet need, respectively. The exception is Factor 2 (Positive Gender Roles, Passive). Progressive male gender role perceptions in the absence of other forms of involvement appears to do little to reduce unmet need among women. At first glance the results for couple’s unmet need look quite different – however, closer examination reveals that the differences in odds ratios of couple’s and women’s unmet need for other than Factors 3 are nominal (although the odds ratio for Factor 1 is not statistically significant in the couple’s unmet need regression). The major difference is for Factor 3 (Active and Direct Involvement). Why might direct and active involvement in family planning have an impact on female but not couple’s unmet need for family planning? The answer lies in Table 1. Two observations emerging from Table 1 are salient. First, the incidence of males alone having demand for family planning within Indonesian couples, which drives the difference between female and couple’s unmet need, is quite small (only about 8% of the ”couple’s sample”). Second, Table 1 indicates that male contraceptive use is relatively invariant across wife-husband combinations of demand for family planning (range 2–4%). It is thus the small size of the relevant sub-population for the calculation of the odds ratio for Factor 3 and the low level of male contraceptive use that account for the results observed in Table 6.

**Table 6:** Relationship between male involvement in family planning for female and couple-level unmet need for family planning

Male Involvement	Female Unmet Need OR [95% CI]	Couple Unmet Need OR [95% CI]
Factor 1	0.899 [0.811–0.995]*	0.915 [0.834–1.004]
Factor 2	1.094 [ 0.995–1.202]	1.083 [0.993–1.181]
Factor 3	0.777 [0.707–0.853]**	1.010 [0.925–1.103]
Factor 4	0.646 [0.602–0.694]**	0.683 [0.638–0.731]**

The qualitative data collected for the study reinforced many of the themes emerging from the quantitative analyses. One theme that emerged clearly from the focus group discussions (FGDs) was the role of male knowledge of family planning:

“Many people don’t know about family planning. It is better if the *posyandu* share information about family planning so that everyone also understands about family planning. My husband already knows about family planning and agrees if I use family planning. I have been using family planning for 20 years, have used injections and switched to using implants until now.” (PL, 39-year-old housewife, Eastern Indonesia).

“My husband knows all the type of methods. But because he’s a man, he’s not aware about it. It’s like he doesn’t want to know because it’s a women’s business. At the beginning after giving birth, I wanted to put an IUD and my husband wondered what an IUD was, its uses and side effects. I explain after browsing. He was offered a vasectomy but didn’t want to.” (AN, 39-year-old female, civil servant, Sumatra/Kalimantan).

“There are still many men who think that family planning programs are a woman’s business, men’s knowledge of contraception is also still low, and there are still many who do not understand the types of male contraception.” (MI, 30-year-old, housewife, Eastern Indonesia).

“Our area received counseling from the midwife/public health center but only the wife, while the men did not participate because they were embarrassed.” (EW, 48-year-old male, farmer Sumatra/Kalimantan).

“In general, there is a massive FP program here, but specifically for male family planning, it is still not well understood for the men themselves…, the socialization program can involve those who have done male family planning as ambassadors.” (B, 35-year-old male employee, Sumatra/Kalimantan.

“In my environment for family planning, most of their wife use contraception. a few still think that family planning only focuses on the wife. The understanding of the man’s family planning is still cannot be understood when in fact it is an alternative part.” (AB, male employee, Sumatra/Kalimantan).

Regarding involvement of men as family planning clients, the limited choices available to men and concerns/negative perceptions about vasectomy were noted by a number of FGD participants.

“In my area rarely men use contraception because there are more women who use it. … There are still few [male users] because the choices are few – only condoms and vasectomy” (MR, 57 year-old male, entrepreneur, Sumatra/Kalimantan).

“Family planning in here common things are often informed by cadre. There are only 2 choices for men.” (MU, 33-year-old male teacher, Eastern Indonesia).

“For male family planning, there is less socialization, indeed there are some obstacles that the information obtained by the community is not very accurate. The second one is still many who rely on the religion which mentions it is haram. …. Many circulate that when men taking family planning their passion is reduced and they are afraid to do a vasectomy. (JL, 54-year-old male, village administrative worker, Sumatra/Kalimantan).

“Maybe there is a need for more socialization of this MOP (i.e., vasectomy). Because there are still many of men and friends say that MOP is the same as being castrated.” (D, 49 year old male, civil servant, Java-Bali).

“People think that after vasectomy, it will be dysfunctional, so they can’t have relationships, or what kind of thing there are still stigmas like that in society. It seems that the strengthening in IEC must really be done and indeed work with experts to carry out education in the community.” (VS, 52-year-old female, program official, Sumatra/Kalimantan).

A major theme in the FGDs was that of perceived gender-assigned responsibility for family planning.

“In theory, the husband’s role is very much needed for the selection of contraceptives. But many applications are surrendered to his wife alone. My husband asked if it was safe for me, it was okay to use it. My husband’s involvement in information about contraceptives is lacking. In Mojokerto, the husband’s participation in male family planning is still rare and is still considered taboo and not an obligation. It’s a woman’s duty.” (NK, age not stated female, lecturer, Java-Bali).

“It seems that because it is the woman who is pregnant, women are more responsible for contraceptive use. But there is also an agreement between husband and wife who is readier and more disciplined to use contraception.” (ANR, 49-year-old female civil servant, Sumatra/Kalimantan).

“While the wife has more choices in the methods, it’s very rare methods for men. Maybe it’s still taboo to talk about the issue of male contraceptives. … Men have high egos, they don’t want to take contraception, so just tell their wives.” (M, male, 48 years old, private sector worker, Sumatra/Kalimantan).

“Family planning matters are left to women…for gender awareness themselves with this paternalistic cultural system in Indonesia…the assumption is actually conservative and still exists in the community if family planning means a kind of castration, no longer manly, no longer able to fulfill needs as husband.” (BI, age not stated female, program manager, Java-Bali).

Other barriers to greater male involvement, particularly as family planning clients, were noted by FGD participants, most notably by program manager participants. Program managers in many locations pointed to distance to health facilities/service providers, limitations in staffing and in particular trained staff trained medical staff on clinical contraceptive services Religious opposition to family planning in general, and of vasectomy (male contraceptive use only in emergency situation when women cannot use contraceptives), was noted in some locations. The continued programmatic focus of promotional and educational efforts primarily on women was also noted.

“For male family planning, it seems that there is no information for male family planning and there is no counseling. So, most of the general public information from PKK, RT, RW are all directed to mothers.” (B, 35-year-old male, civil servant, Java-Bali).

## Discussion

Direct male involvement in family planning as family planning clients in Indonesia remains limited – per the 2017 IDHS data, male-controlled contraceptive methods accounted for only about 8% of the method mix of married couples who were using a contraceptive method at the time of the survey. However, our analyses indicate that Indonesian men engage in family planning matters in several other ways that have material consequences for unmet need for family planning among both women and couples. Indeed, the factor analyses undertaken for the study revealed four [4] underlying independent “principal components” or “dimensions” of male family planning involvement in Indonesia, only one of which had to do with involvement as family planning clients.

In terms of impact on unmet need for family planning among both females and couples, male approval of family planning appears to be the most powerful form of male involvement (even if not supplemented by further involvement). Given the low participation of males as contraceptive users, the primary mechanism by which male approval translates into lower unmet need is by enabling females to take the lead in realizing intentions with regard to the number and timing of future pregnancies. Although males do facilitate female use of contraception to some extent via communications and participation in contraceptive decision making and male IDHS respondents professed gender-positive attitudes toward responsibility for family planning generally and sterilization specifically, the qualitative data suggest that family planning continues to be seen by large segments of Indonesian society as being the responsibility of women, a perception reinforced by the perception that national family planning program efforts target women. Given male approval, Indonesian women appear to respond to their own desires to limit or space future pregnancies as well as those of their husbands, at least sometimes even when their husband’s desires do not align with their own.

In its Strategic Plan for 2020–2024 [44], the National Population and Family Planning Board (BKKBN) is focusing on two of three types of male involvement articulated by Greene et al. (2006) – men as family planning clients and men as agents of change. Although the BKKBN also recognizes the importance of men as agents of change as Male Family Planning Groups are envisioned as a key mechanism for advancing the male involvement agenda, the primary focus appears to be on men as family planning clients given the priority assigned to significantly increasing the uptake of vasectomy to reach 5.7% prevalence among married couples using contraceptives by 2024. However, rapidly increasing vasectomy prevalence is challenging in Indonesia, as it has proven to be in other countries [45], given Indonesia’s sociocultural and supply environment contexts and the level of misinformation about vasectomy circulating in society at large. Reaching the target will thus require concerted efforts on several fronts. Even if the BKKBN target of increasing the proportion of married couples relying on vasectomy to avoid unwanted or mistimed pregnancies to 5.7% by 2024 is achieved, the impact is likely to be modest. To magnify the impact, increased use of male condoms might also be promoted, and more importantly greater attention might be assigned to a third type of male involvement– men’s roles as partners; that is, on how men can help facilitate contraceptive use by females, the receipt of maternal and child health services, and male involvement as fathers in the provision of childcare.

The global evidence suggests that Indonesia may benefit from a more holistic approach that goes beyond a narrow focus on men and family planning. The expansion and culturally nuanced implementation of “Gender Transformative Programming” (GTP) or similar/derivative approaches would seem a logical way forward. The Partnership for Maternal, Newborn & Child Health (PMNCH) defines GTP as an approach that “recognizes and addresses the individual, institutional and cultural dynamics that influence the behaviors and vulnerabilities of men and women [46]. The evidence reviewed in the 2013 UNFPA/PMNCH “Knowledge Summary” suggests that male roles in Reproductive, Maternal, Newborn, and Child Health (RMNCH) services that can be reinforced via gender transformative approaches include shared responsibility for family planning, contraception, and prevention of STIs; helping pregnant women stay healthy and deliver their babies safely; and engaging in responsible fatherhood and caregiving of children.

However, the available evidence on suggests that gender transformative efforts must go beyond men to also include service providers. Evidence from studies of determinants of male involvement in MCH services in sub-Saharan Africa showed that health providers played a key role in affecting male involvement in Prevention of Mother-to-Child Transmission (PMTCT) and ostensibly for broader RMNCH issues [28, 46]. Among the factors identified as discouraging male involvement were harsh behavior/language from service providers and service provider perspectives that taking care of participating male partners is considered an additional burden in settings where health services providers are overworked, stressed, and working with severely limited resources. Based upon these findings, which resonate with comments made by male FGD participants in the present study, alternative service models targeted at men may be needed, including [1] use of appointment systems to reduce male (and female) “opportunity costs”, [2] broadening service hours to evenings and weekends, and [3] use of alternative, more “male friendly” venues not traditionally associated with health care such as religious establishments, workplaces, and other sites where males tend to gather.

The results of the present study provide some clues as to priority targeting. In the aggregate, the results of the analyses of correlates of male involvement in family planning suggest that it is age, education, residence on Java-Bali vs. outer islands), and knowledge of contraceptive methods that distinguish men with higher and lower levels of involvement. The finding with regard to education is nearly universal in earlier studies. Based upon these findings, the priority targets for efforts to increase involvement in family planning should be younger, less well-educated men residing on islands other than Java-Bali and men who are not supportive of family planning. However, as the impact of efforts to increase male involvement in family planning will depend upon both changes in broader social norms and a focused response by service providers a national initiative that will also target community and religious leaders and health service providers will be needed. All efforts to involve men should be designed using gender transformational principles.

An enabling policy environment is essential to making significant progress in more fully engaging males. A recent assessment of policy barriers to and enablers of male engagement in family planning based upon a male involvement framework developed for the HP + Project found that of the 26 countries assessed, only four were identified as having a strong enabling environment with comprehensive approaches to engage men as family planning clients, supportive partners, and agents of change, and included strong provisions to address the principles of male engagement identified in the framework [47]. The majority of countries assessed (14 out of 26) were categorized as having average enabling environments, while the remaining eight countries were classified as having weak enabling environments. A systematic self-assessment of the Indonesian policy environment for male involvement in family planning by the BKKBN would be a useful starting point in seeking avenues via which to further expand male involvement.

Recent years have witnessed the development of a number of frameworks and sets of guidelines for advancing the male involvement in family planning agenda [30, 33, 47–50]. Collectively, these efforts produce a set of basic of principles for male engagement in family planning. These include the need to [1] use age-appropriate, life-stage approaches tailored to cultural contexts, [2] implement multisectoral and integrated programs, [3] respect women’s autonomy while meeting men’s and boys’ needs, [4] ensure that all initiatives are rights-based and entail voluntary participation, [5] engage men and boys from a positive perspective, [6] emphasize that choices as to numbers and timing of children have long-term impacts on their own lives, and [7] ensure the availability of data to track differential impacts of family planning policies and programs by gender. The available evidence also suggests that multi-theme vs. single-issue interventions tend to be more effective in realizing behavior change, particularly those that combine community outreach, mobilization, and mass-media campaigns with group education [46].

Beyond the desirability of increasing gender equality in all of life’s domains, it might thus be asked why increasing male involvement is important for the Indonesian national family planning program given its level of success with current levels of involvement of men? There are at least two reasons. First, despite the success of the Indonesian national family planning program, significant weaknesses remain – relatively high levels of unmet need for family planning measured at either the female or couples’ levels, relatively high rates of contraceptive discontinuation, and non-trivial rates of induced abortion [41, 45, 51]. It may simply be the case that the national program has plateaued if women continue to bear the responsibility for family planning without more active involvement from their spouses. Second, the present study focused on male involvement in family planning, a program arena in which Indonesia has been relatively successful. Indonesia has been less successful in the maternal health arena, with maternal mortality ratios that are significantly higher than might be expected given the country’s level of economic and health system development – 305 maternal deaths per 100,000 live births [52]. Here, the meta-analysis results of Yargawa and Leonardi-Bee [25] showing significant positive effects of male involvement on improved rates of utilization of maternal health services and lower rates of maternal depression during pregnancy and postpartum are instructive. This is especially significant as a recent study showed that poor and near-poor, urban Indonesian women lagged significantly behind non-poor urban women in terms of the quantity and quality of maternal health services received in connection with recent pregnancies [53]. Greater male involvement as partners that results in pregnant women receiving adequate maternal health services might well be a “game changer” in Indonesia’s long-standing struggle with high maternal mortality.

## Conclusions

Although male disapproval of family planning is limited in Indonesia, the predominant form of male involvement remains passive approval, resulting in women bearing most of the responsibility for realizing couple reproductive aspirations. Further actions that engage men as clients and as partners, and address broader gender issues are needed given the prevailing social norms surrounding family planning. Transformative programming must extend beyond men to also target health service providers, community and religious leaders in order to provide an enabling environment for transformative change.

## Data Availability

The quantitative raw data for this study are available online in the DHS website (https://dhsprogram.com) and BKKBN national programmatic data by applying through the website. The authors lack the authority to upload the data to other repositories.

## Abbreviations

BKKBN: Badan Kependudukan dan Keluarga Berencana Nasional (National Population and Family Planning Board)
CPR: Contraceptive Prevalence Rate
DFA: Discriminant Function Analysis
FGD: Focus Group Discussion
FP: Family Planning
GTP: Gender Transformative Programming
HIP: High Impact Practices
ICPD: International Conference on Population and Development in Cairo
IUD: Intrauterine Device
IDHS: Indonesian Demographic and Health Survey
NFP: National Family Planning Program
PKK: Pemberdayaan Kesejahteraan Keluarga (Family Welfare Program)
PLKB: Penyuluh Lapangan Keluarga Berencana (Community Family Planning Worker)
PMNCH: Partnership for Maternal, Newborn and Child Health
PMTCT: Prevention of Mother-to-Child Transmission
RMNCH: Reproductive, Maternal, Newborn, and Child Health
SDG: Sustainable Development Goals (SDGs)
STI: Sexual Transmitted Infection
UNFPA: United Nations Population Fund
WHO: World Health Organization
